# Systemic Delivery of siRNA Down Regulates Brain Prion Protein and Ameliorates Neuropathology in Prion Disorder

**DOI:** 10.1371/journal.pone.0088797

**Published:** 2014-02-14

**Authors:** Sylvain Lehmann, Aroa Relano-Gines, Sarah Resina, Elsa Brillaud, Danielle Casanova, Charles Vincent, Claire Hamela, Sophie Poupeau, Mathieu Laffont, Audrey Gabelle, Constance Delaby, Maxime Belondrade, Jacques-Damien Arnaud, Maria-Teresa Alvarez, Jean-Claude Maurel, Patrick Maurel, Carole Crozet

**Affiliations:** 1 Institut de Médecine Régénératrice et de Biothérapie (I.M.R.B.), Physiopathologie, diagnostic et thérapie cellulaire des affections neurodégénératives –Institut National de la Santé et de la Recherche Médicale Université Montpellier 1 U1040 Centre Hospitalo-Universitaire de Montpellier, Université Montpellier 1, Montpellier, France; 2 Institut de Génétique Humaine, Centre National de la Recherche Scientifique- UPR1142, Montpellier, France; 3 Medesis Pharma SA, Baillargues, France; 4 Université Montpellier2, Montpellier, France; Dulbecco Telethon Institute and Mario Negri Institute for Pharmacological Research, Italy

## Abstract

One of the main challenges for neurodegenerative disorders that are principally incurable is the development of new therapeutic strategies, which raises important medical, scientific and societal issues. Creutzfeldt-Jakob diseases are rare neurodegenerative fatal disorders which today remain incurable. The objective of this study was to evaluate the efficacy of the down-regulation of the prion protein (PrP) expression using siRNA delivered by, a water-in-oil microemulsion, as a therapeutic candidate in a preclinical study. After 12 days rectal mucosa administration of Aonys/PrP-siRNA in mice, we observed a decrease of about 28% of the brain PrP^C^ level. The effect of Aonys/PrP-siRNA was then evaluated on prion infected mice. Several mice presented a delay in the incubation and survival time compared to the control groups and a significant impact was observed on astrocyte reaction and neuronal survival in the PrP-siRNA treated groups. These results suggest that a new therapeutic scheme based an innovative delivery system of PrP-siRNA can be envisioned in prion disorders.

## Introduction

The development of new therapeutic strategies for neurodegenerative disorders is a major medical, scientific and societal issue. Among human pathologies, Creutzfeldt-Jakob diseases are rare neurodegenerative fatal disorders which today remain incurable [1]. They belong to prion diseases also called transmissible spongiform encephalopathies (TSE). TSE are characterized by neurodegeneration, rapid neuronal cell death, vacuolisation and gliosis [1], [2]. They affect both humans and animals and have a long incubation time before an insidious progression of the disease. The evolution towards dementia is rapidly fatal. The exact nature of the infectious agent responsible for these diseases remains source of debate and is the subject of many studies. It appears to be associated with PrP^Sc^, which accumulates in the brain of the host [3]. This protein corresponds to an abnormal isoform, resulting from the post-translational change of the host-encoded PrP^C^ protein. PrP^Sc^ presents specific characteristics such as the formation of aggregates and a high resistance to hydrolysis by proteases [3]. Many investigations on TSE have been performed using fully characterized preclinical models of infected mice, well suited for the validation of new therapeutic approaches. Many studies performed in the last twenty years have demonstrated the requirement for endogenous PrP expression in the development of a TSE [4], suggesting that inhibiting PrP expression could constitute a good strategy to interfere with prion propagation. Indeed, transgenic mice in which the *Prnp* gene has been knocked-out do not propagate prion infection and do not develop a TSE following inoculation with scrapie prions [4], [5]. In addition, conditional knockout of PrP solely in neurons, approximately 8 weeks after intraperitoneal (i.p.) prion infection, not only prevents disease but also reverses early spongiform changes [6]. Recently, extinguishing the expression of the prion protein *Prnp* gene using posttranslational gene silencing mediated by RNA interference (RNAi) was shown to be promising in the search for prion disorder treatments. RNAi is a mechanism that inhibits gene expression by hindering the transcription of specific genes in a wide variety of eukaryotic organisms [7], [8]. Lentiviral vector-mediated PrP shRNA (ShortHairpin RNA), was able to significantly reduce neuronal PrP^C^ expression. By removing the substrate for conversion into PrP^Sc^, they were able to suppress PrP^Sc^ accumulation in the treated cells resulting in a strong increase in the incubation time, a rescue of neuronal damage in the treated area and a strong improvement of some cognitive functions [9]–[13].

Promising therapeutic approaches aiming to block the production of PrP^Sc^ based on PrP RNA interference without genetic manipulation constitutes therefore a real challenge. Recently, in the search for drug delivery vectors, Medesis Pharma has developed a water-in-oil microemulsion technology called Aonys. This technology allows the intracellular delivery of water-soluble active pharmaceutical ingredients (API) including siRNAs, peptides and inorganic metal salts [14] in all tissues of the organism. The delivery steps involve : i) absorption of the API containing formulation through mucosa and combination with apolipoproteins, most likely pre β-HDL [15], [16]; ii) secretion into the lymphatic system; iii) systemic circulation of API protected into VHDL/HDL; iv) tissue distribution including blood brain barrier crossing and cellular uptake via SR-B1 receptor-mediated transport [17]. The main advantages of Aonys over standard drug formulations are: i) protection of API during distribution phase, ii) enhanced cellular delivery, iii) non-invasive route of administration. While the use of invasive routes of administration (intravenous, sub-cutaneous…) is undesirable in chronic diseases where treatments can last months or years, Aonys offers a non-invasive per mucosal (rectal, buccal) delivery route for biomolecules.

In this context, the general objective of this study was to evaluate the efficacy of PrP-siRNA delivered by Aonys. After 12 day rectal mucosa administration of PrP-siRNA in wild-type mice, we observed a decrease of about 18% in PrP mRNA levels while brain PrP^C^ was also decreased by 28%. The effect of PrP-siRNA was then similarly evaluated on ME7 murine prion infected mice. Several mice presented a delay in the incubation time and improvements in survival compared to the control groups, but there was no global statistical significant difference. However, positive impacts on the astrocyte reaction and neuronal survival were observed in the PrP-siRNA treated groups.

## Results

### Effect of Aonys/PrP-siRNA on *Prnp* Expression in Naive Mice

We first tested the capacity of Aonys/PrP-siRNA to inhibit *PrnP* expression in the brain of naive wild-type mice. Two doses of PrP^C^ siRNA were compared: 300 (n = 10) and 600 µg (n = 10) siRNA/kg. Control animals were treated with Aonys vehicle (n = 10) or with Aonys/scrambled-siRNA (n = 10). Animals were treated for 12 days (8 administrations, see table 1) via rectal mucosal administration. On day 13 the mice were sacrificed, brains were collected and PrP^C^ expression levels were assessed by both western-blot,ELISA, and RT-Q-PCR.

**Table 1:** Chronology of the in vivo phase.

*Days*	*1*	*2*	*3*	*4*	*5*	*6*	*7*	*8*	*9*	*10*	*11*	*12*	*13*
*Events*	*Adm.*	*Adm.*			*Adm.*	*Adm.*	*Adm.*	*Adm.*	*Adm.*			*Adm.*	*sacrifice*

**Figure 1:**
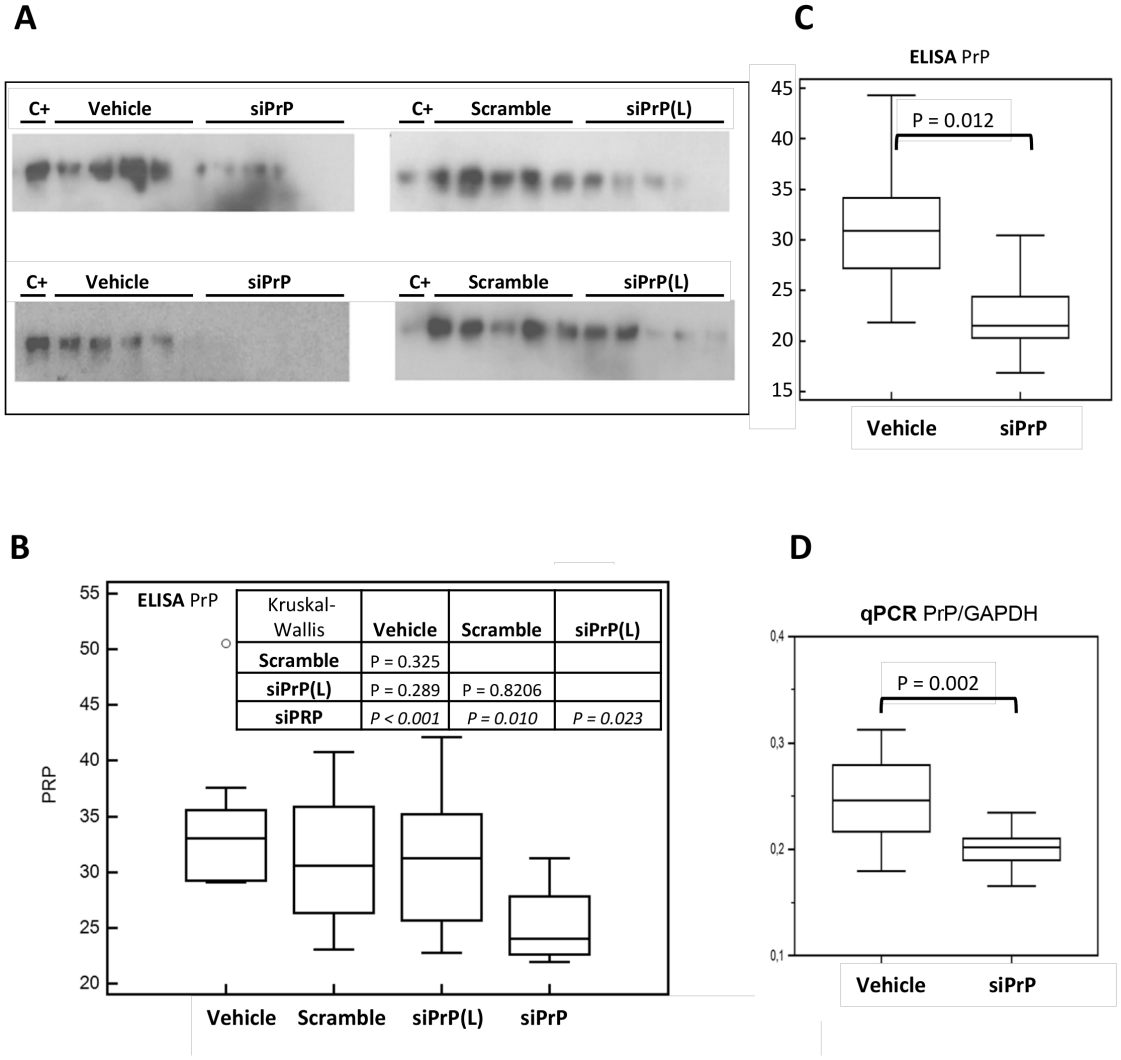
In vivo PrP^C^ expression in AONYS/siRNA-treated mice. Panel A. Brain homogenates of mice treated for 12(see Table 1) with two doses of PrP^C^ siRNA (Aonys/siPrP 300 (L) and 600 µg/kg), the vehicle only and scrambled-siRNA, were analysed for PrP^C^ accumulation by Western blot. The C+ lane corresponds to a brain homogenate of untreated mice. A decrease of PrP^C^ was apparent in siPrP treated mice. However there was a relative variability in detection in relation with the low linearity of the western blot. Panel B. Samples in A were analysed using ELISA for quantitation of PrP^C^. Graphic results of normalized PrP^C^ were presented as medians and interquartile ranges. Statistical analysis (Kruskal-Wallis test) was used to evaluate the significance of the difference between the groups. The siPrP group (600 µg/kg) presents a significant reduction of PrP^C^ levels. Panels C and D. Additional experiments were carried out (10 mice/groups) in which mice were treated or not with Aonys/PrP^C^ siRNA at 600 µg/kg. Panel C. PrP^C^ ELISA. Panel D. RT-Q-PCR quantitation (light Cycler) of PrP and GAPDH in Graphic results were presented as medians and interquartile ranges. Statistical analysis (Kruskal-Wallis test) was used to evaluate the significance of the difference between the groups. The siPrP group presents a significant reduction of both PrP^C^ protein and RNA levels.

PrP levels evaluated by western-blot were lower in the groups treated with Aonys/PrP-siRNA (Figure 1A). ELISA confirmed these results which were most important with the highest dose level of PrP-siRNA (Figure 1B,C) for which a significant 28% decrease (p = 0.01 vs scramble) was observed. These results were correlated with a 17.6% decreased level of PrP mRNA determined by RT-Q-PCR in independent additional experiments (Figure 1D).

### Effect of Aonys/PrP-siRNA on Prion Infected Mice

Our second objective was to evaluate the efficacy of PrP-siRNA delivered by Aonys, on such disease parameters as incubation/survival time, astrocytosis, neuronal survival, and vacuolisation. For this purpose, we used C57BL/6J mice intracerebrally inoculated at day 1 with ME7 prion infected brain homogenates (20 µL of 1% brain homogenate). Treatment was initiated at day 90, and continued until the death of animals.

### Impact on Incubation Period and Survival Time

**Figure 2:**
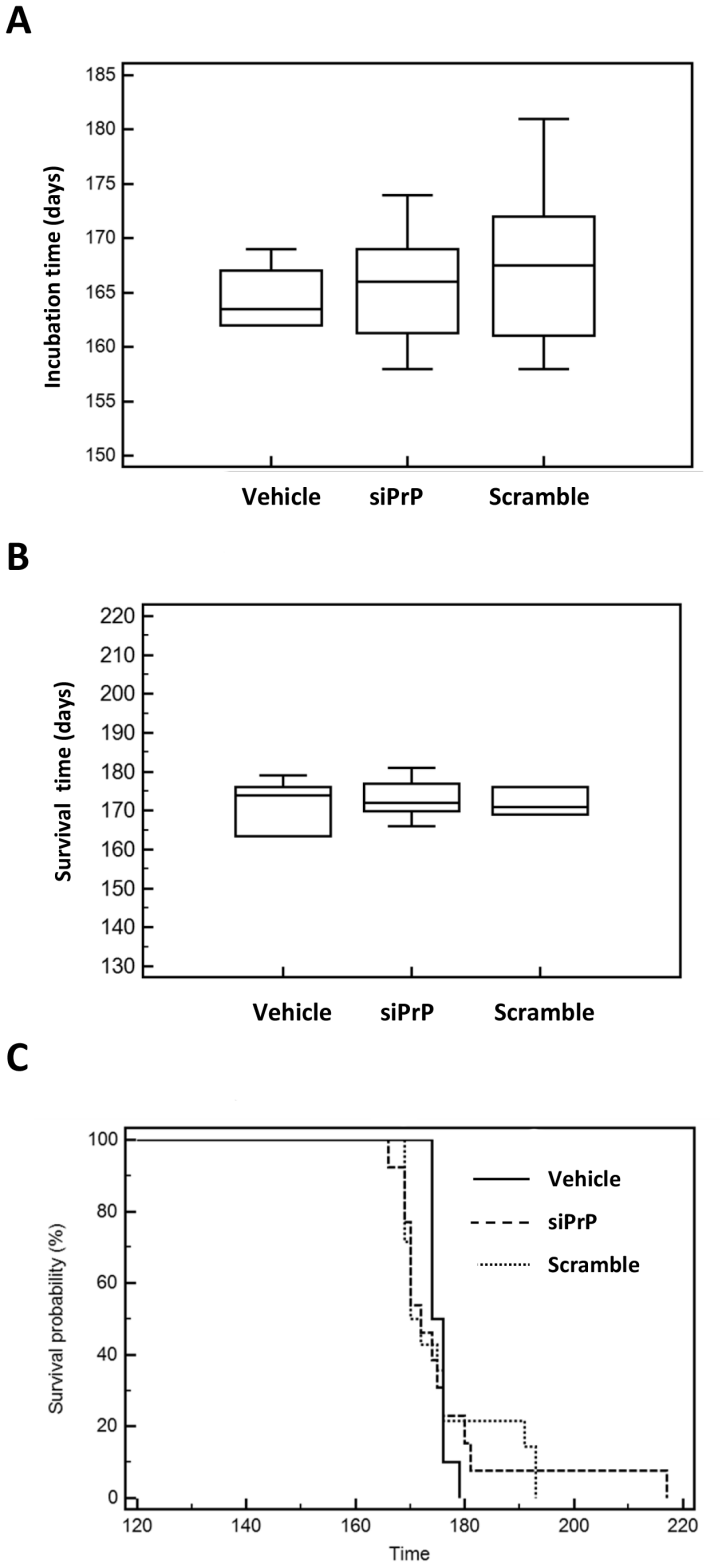
Impact of siRNA on incubation and survival time. Panel A. The incubation time in scrapie infected mice based on the presence of at least three clinical signs (among: waddling gait, flattened back, rough coat, sticky eye discharge, weight loss, very jumpy, hunched, incontinence) was plotted as medians and interquartile ranges in the three groups: Aonys/PrP^C^ siRNA (siPrP 600 µg/kg), Aonys vehicle only and Aonys/scrambled-siRNA. The survival times of the three groups of infected mice were plotted as medians and interquartile ranges (Panel B.) and Kaplan-Meier survival curves (Panel C.). No statistical difference was observed between each group neither for the incubation period nor the survival time.

Each animal was observed at least once per day for mortality and morbidity before treatment and throughout the treatment period. The appearance of a list of clinical signs was checked and recorded to allow the determination of the incubation period that corresponds to the presence of at least three clinical signs. Even though we observed a slight increase of the incubation and survival time for the siRNA groups (PrP-siRNA and scrambled-siRNA), no significant difference was observed either for the incubation period (Figure 2A) or the survival time (Figure 2B, C). However the spread of incubation time and survival time was larger in the siRNA treated group (166–217 days for PrP-siRNA, 169–193 days for scrambled-siRNA) compared to the vehicle treated group (174–179 days) (Figure 2C).

### Effect on PrP^Sc^ Accumulation

**Figure 3:**
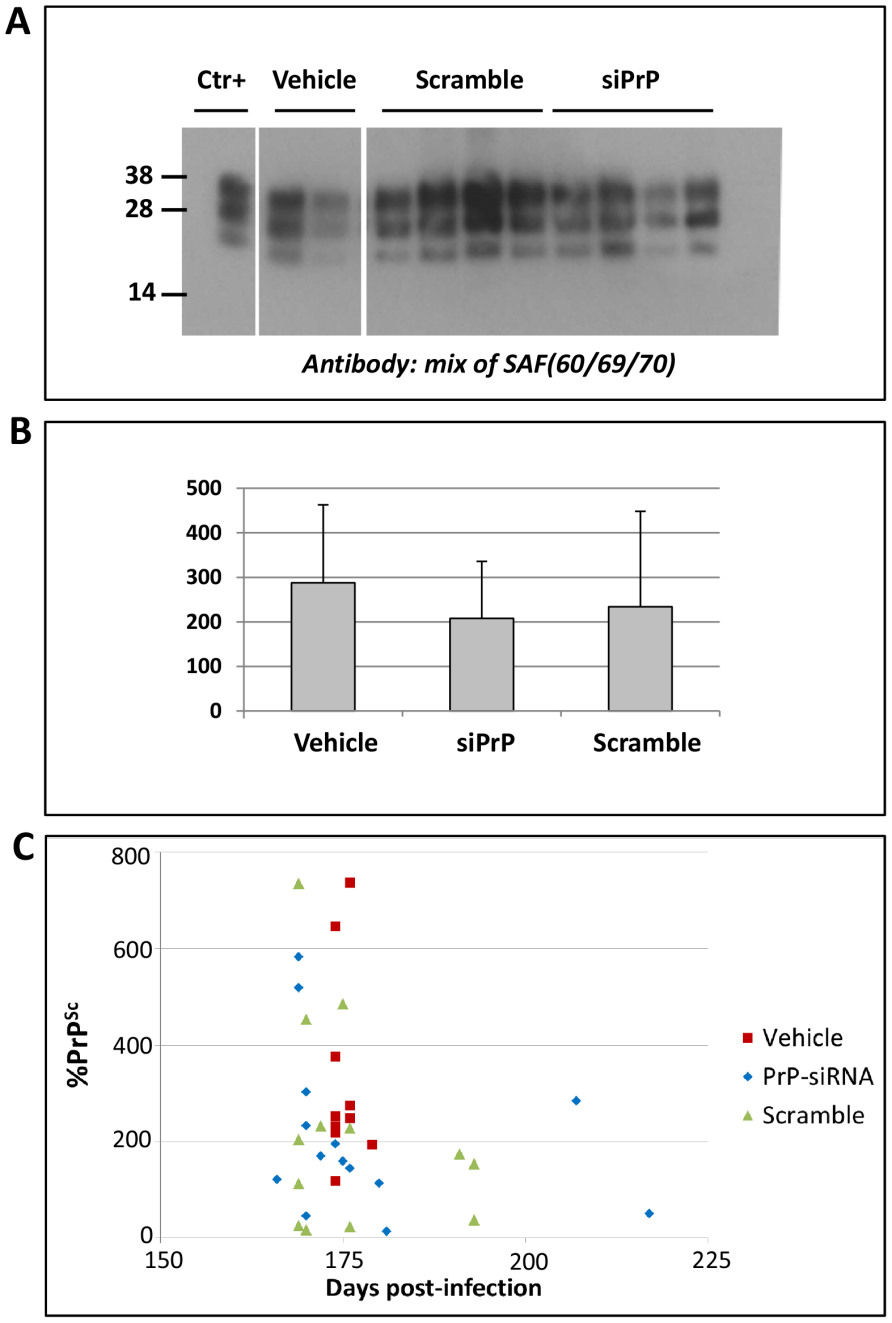
Impact of siRNA on brain PrP^Sc^ content. Western blot analysis of PrP^Sc^ in mouse brain lysates (Panel A.). Only representative samples of the mouse population are presented here. The classical ME7 PrP^Sc^ bands were present at various intensity levels. The Ctr+ lane corresponds to a brain homogenate of ME7 scrapie mice loaded as a standard in every blot. Quantitation of the signal (Panel B.) normalized in each blot with respect to the Ctr+ sample showed no significant difference between groups. (Panel C.) Individual PrP^Sc^ levels and survival times.

The prion infection of the mice was confirmed by detecting PrP^Sc^ accumulation by western blot analysis of proteinase K digested brain homogenates. There was some variability between the different samples with a slight decrease for both the scrambled and PrP-siRNA treated mice (Figure 3A) but no statistically significant difference was observed (Figure 3B).

The representation of individual levels of PrP^Sc^ and survival time (Figure 3C) did not reveal a correlation between them. This could be explained by the fact that all brains have been collected at the terminal stage of the disease were PrP^Sc^ is known to plateau.

### Effect on Vacuolization, Astrocytosis and Neuronal Count

**Figure 4:**
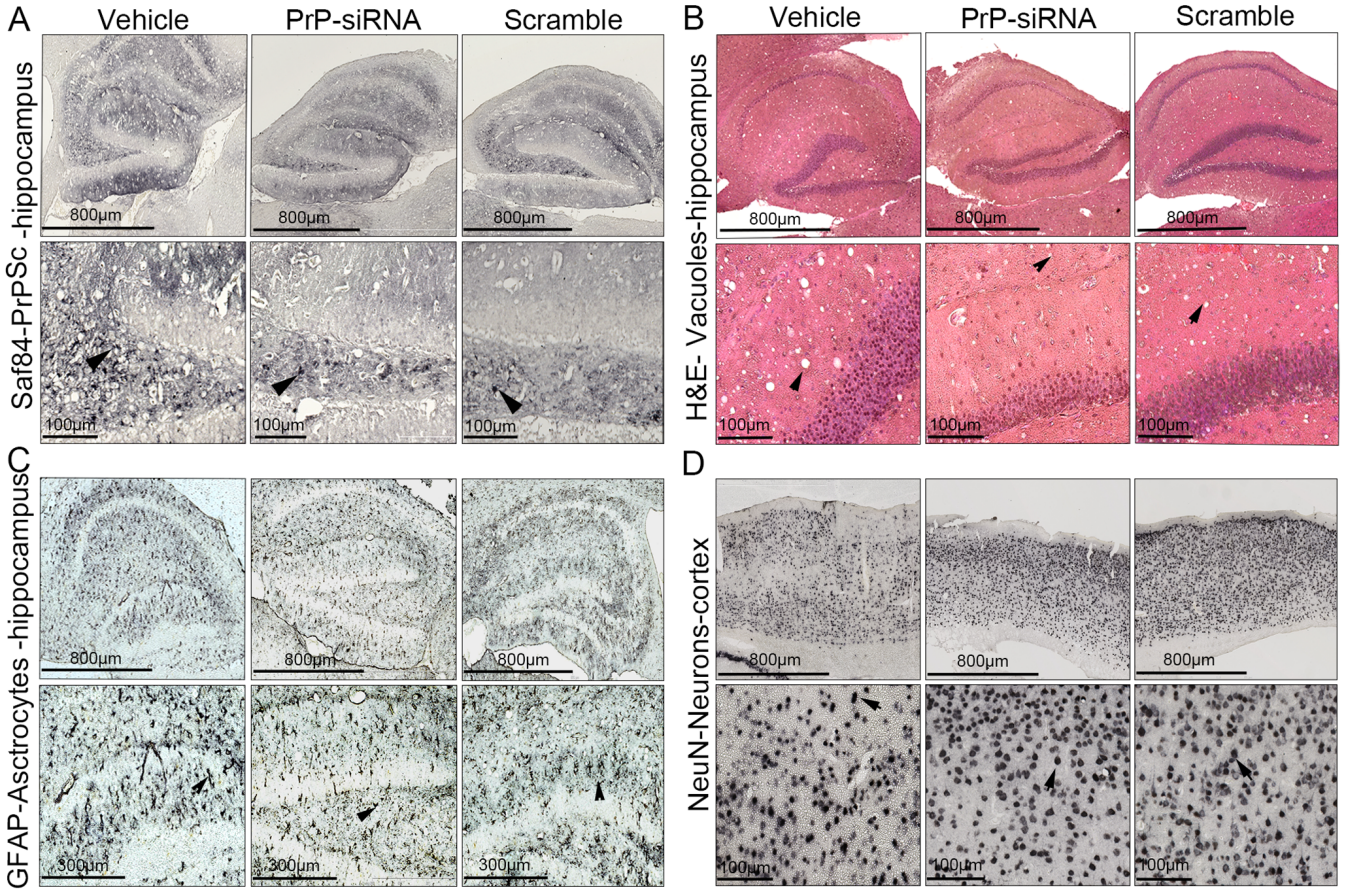
Histological and Immunohistolochemical illustration. Panel A. PrP^Sc^ immunostaining was observed in all the mice (arrow). Panel B. Vacuolar lesions revealed after Hematoxylin & Eosin staining were apparent in all conditions. Immunohistochemical detections of the GFAP astrocyte marker (Panel C) and the neuronal marker NeuN were also performed (Panel D).

**Figure 5:**
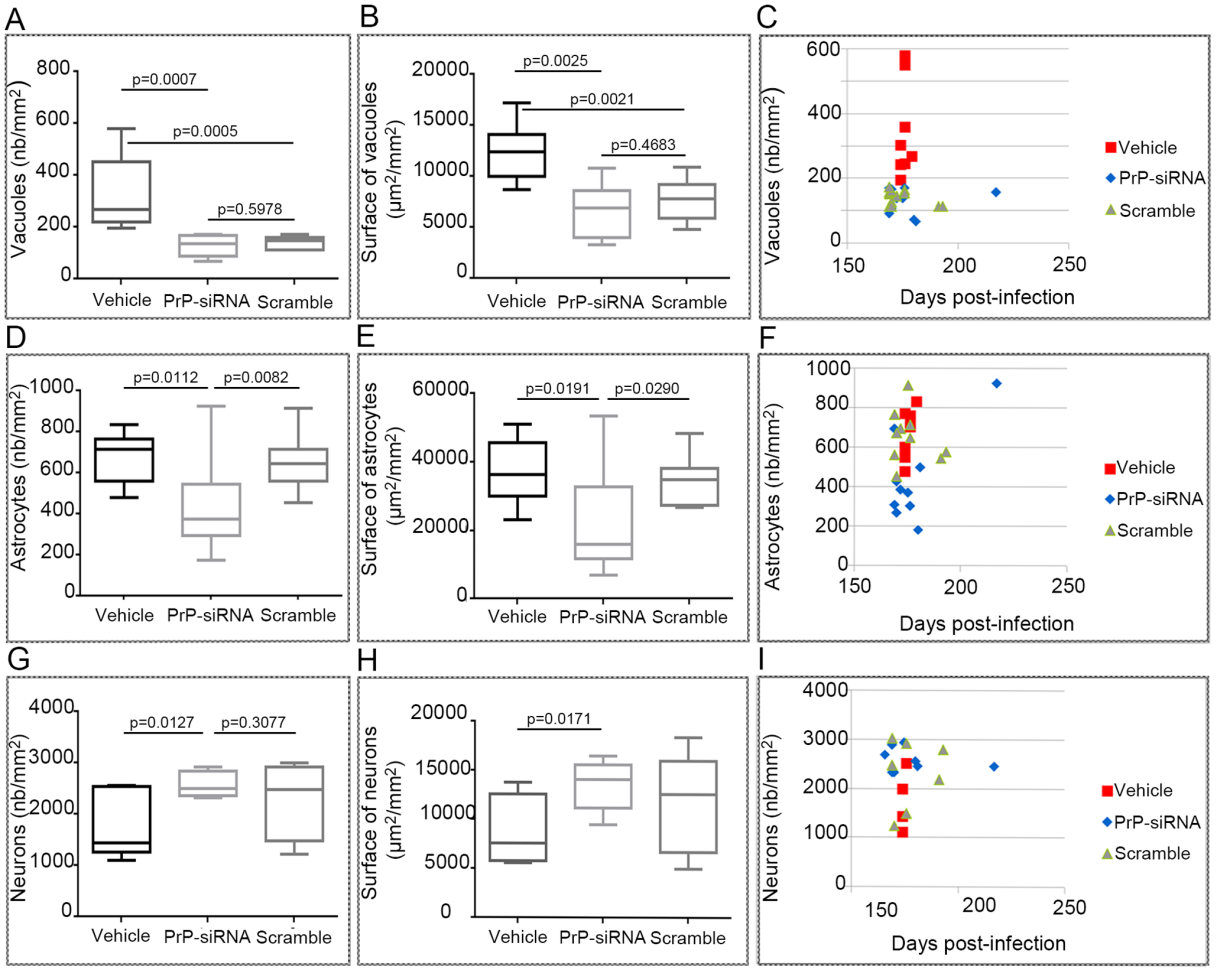
Histological and Immunohistolochemical data analysis. Panels A,B. Quantitation of vacuolar lesions after Hematoxylin & Eosin staining (figure 4) revealed that siRNA have a slight impact. Panels D,E. Quantitation of astrocyte based on the GFAP immunostaining (figure 4) showed significant differences between the Aonys/PrP-siRNA treated group and the two control groups (vehicle and scrambled-siRNA). Panels G,H. Quantitation of neurons based on the neuronal marker NeuN (figure 4) revealed statistical differences between mice treated with PrP-siRNA and vehicle. Panels C,F,I. Data of individual levels of vacuolisation, NeuN or GFAP counts as a function of survival time did not show any correlation.

Half of each brain was collected for histological and immunohistochemical analysis on paraffin embedded 5 µm thickness sections. PrP^Sc^ immunostaining was performed using SAF84 antibody (Figure 4A). We observed typical PrP^Sc^ immunostaining patterns in each brain analysed: diffuse, ponctiform or amyloid deposits. Hematoxyline & Eosine staining was performed to detect vacuolar lesions (Figure 4B). Vacuole number (nb/mm^2^) and surface (µm^2^/mm^2^) were determined in the hippocampus. When compared to the vehicle control group, vacuole number and surface were significantly lower in the two siRNA treated groups (Figure 5A,B). The level of vacuolization was lower in the PrP-siRNA treated group compared to the scrambled siRNA treated group but without any statistical significance. Representation of individual number of vacuoles and survival times, while showing clusters of siRNA-treated and vehicle groups, did not indicate any correlation between vacuolization and survival time (Figure 5C). Immunohistochemical analysis of the GFAP astrocyte marker was performed to assess whether this particular hallmark of the disease was modified during the treatment (Figure 4C). We evaluated both number (nb/mm2) and surface (µm2/mm2) of astrocytes in the hippocampus. Significant differences (Figures 5D, E) were obtained between the PrP-siRNA treated group and the two control groups (vehicle and scrambled-siRNA). Immunohistochemical analysis of the NeuN neuronal marker was also performed to assess whether neuronal survival in a cortex area was improved (Figure 4D). Significant differences (Figures 5G,H) were also obtained between the PrP-siRNA treated group and the vehicle group. Even though there were more neurons in the cortex area from mice treated with PrP-siRNA compared to mice treated with the scrambled-siRNA, the difference was not significant. Despite clustering between groups, no correlation was observed between astrocyte, vacuole or neuron counts and survival times (Figures 5C,F,I).

### Cytokines Production

**Figure 6:**
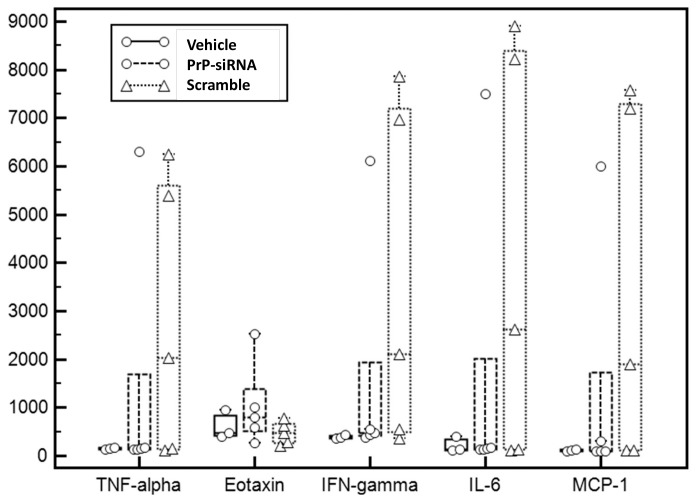
Cytokines analysis. Graphic representation plotted as medians and interquartile ranges of selected blood cytokine levels in the three mice groups (see table 2 for full data). Note that outliners characterized by very high cytokine levels were present in the siRNA groups only.

In order to assess whether some immune reaction was promoted by the use of RNA interference, pro-inflammatory cytokine levels were determined in the blood of several animals by a protein array technique (see table 2). Statistical analysis of the group PrP-siRNA and scrambled-siRNA revealed no significant difference for the different probes (Figure 6). However at the individual level, some mice of the scrambled-siRNA treated group exhibited a high level of TNF-alpha, Eotaxin, IFN-gamma, IL6 and MCP-1 suggesting a high inflammatory status in these animals which was not correlated with the survival time (data not shown).

**Table 2:** Cytokine results.

	Vehicule (n = 3)		siRNA (n = 5)		Scramble (n = 5)	
	Median	25–75 P	Median	25–75 P	Median	25–75 P
Thrombopoietin	756	707–780	717	624–959	1506	769–1658
VEGF	27	26–29	30	21–61	53	34–71
TIMP-1	524	439–873	504	300–1069	880	394–1814
TIMP-2	722	677–787	802	692–889	1152	779–1317
TNF-alpha	152	134–167	149	139–1699	2035	137–5601
bFGF	439	418–490	476	439–537	571	457–1026
Eotaxin	478	414–838	806	515–1379	475	265–669
Fas Ligand	15	10–23	15	10–17	20	18–51
G-CSF	57	46–351	79	54–292	112	52–290
GM-CSF	102	90–131	111	97–1053	1740	85–5450
IFN-gamma	370	368–427	468	426–1938	2108	498–7189
IGF-II	160	156–194	161	142–217	350	170–392
IL-1 alpha	82	65–99	70	62–130	158	67–439
IL-12 p40/p70	137	136–170	158	138–438	570	181–1703
IL-12 p70	214	173–215	206	1681772	1879	168–5765
IL-13	34	27–45	42	34–73	46	32–102
IL-1beta	52	38–71	43	30–119	33	29–103
IL-6	139	122–335	151	133–2004	2621	129–8388
IL-9	482	465–521	525	490–1337	1687	542–4002
Leptin	77	45–95	72	47–72	134	83–221
M-CSF	89	83–99	87	81–206	207	79–541
MCP-1	115	101–134	98	941721	1894	114–7294
MIG	19	19–21	22	13–92	35	19–134

## Discussion

For many years, PrP^C^ or PrP^Sc^ entities have been the target of several anti-prion therapeutic strategies. Identification of many compounds in prion cell culture models failed to promote pathological amelioration in prion animal models [18]. Recently, gene therapy using dominant negative PrP mutant or PrP-shRNA [11], [19] as well as a cell therapy approach developed by our laboratory [20], [21] have lead to an increase of the incubation period and a decrease in astrocytosis and vacuolization parameters. We therefore proposed to test a new therapeutic scheme using this new drug delivery technology named Aonys to deliver PrP-siRNA, first in wild type mice and then in prion infected mice. Aonys comprises exclusively pharmacopeia and GRAS listed ingredients. It has been shown to be safe in pre-clinical and phase 1 clinical studies, and represents an alternative to intravenous or subcutaneous administration of biopharmaceuticals, enabling non-invasive permucosal (rectal or buccal) delivery of biomolecules. In our paradigm, the rectal route was selected because buccal permucosal administration, the intended route of administration in the clinic, is difficult in mouse, requiring animal anesthesia.

In our experimental conditions, the delivery of PrP-siRNA during 12 days promoted a decrease of 28% of PrP^C^ in brain which represents a slightly better decrease compared to what had been previously observed by Pulford et al. [22] using Liposome-PrP-siRNA-Peptide Complexes. This result also confirmed that the daily mucosa rectal delivery of Aonys/PrP-siRNA was not only well tolerated but was also able to cross the blood brain barrier as has been previously shown in paralleled experiments carried out for pharmacokinetics, tissue distribution and pharmacodynamics with different API including lithium, siRNAs and peptides ( [14] and unpublished data). Indeed, the API, irrespective of its nature, reaches a maximum concentration in the blood approximately 3 hours after administration, and crosses the blood brain barrier as revealed by direct analysis in brain tissue. The dose range tested in this first experiment was very limited (300 µg and 600 µg). The reason for this lies in the fact that the dose of 600 µg is the maximum that can be used in order to remain in the domain of stable, isotropic liquid, translucid microemulsion (as determined from the phase diagram), a mandatory condition for efficient permucosal delivery. We also did not test lower doses of siRNA (i.e. 100 µg) because we know from other studies that this low concentration do not result in a significant delivery following our route of administration. The results obtained with wild-type mice prompted us to test the efficacy of Aonys/PrP-siRNA in a preclinical model of prion disorder. The disease is characterised by vacuolization and neuronal loss, typically with a bilateral, symmetrical distribution as well as astrocyte proliferation and prion protein accumulation. We decided to begin the treatment at 90 days post-infection. This is considered as a late treatment in which the clinical cognitive defect is mimicked. When survival and incubation time were considered, we observed no difference between the groups. However the spread of incubation and survival time was higher in the siRNA-treated groups (166–217 days for PrP-siRNA, 169–193 days for scrambled-siRNA) compared to the vehicle-treated group (174–179 days). This is likely due to low and variable brain distribution of siRNA after permucosal administration (JC Maurel, personal communication). It is also important to note that in the PrP-siRNA-treated groups some mice died a long time after those of control group. This corresponds for these mice to an improvement of the survival of 15.6 to 21.2%. The brain levels of PrP^Sc^ assessed by western blot in the PrP-siRNA-treated mice were slightly lower than those of control mice but this difference was not significant. The fact that mice were sacrificed when they exhibited a range of clinical signs likely accounts for this observation. The anatomopathologic examination of all brains revealed statistically significant results for the number and the surface of vacuoles in the PrP and scrambled siRNA-treated brains compared to the vehicle treated brains. NeuN immunhistochemical analysis showed a better neuronal survival in mice treated with the PrP-siRNA compared to both control without reaching the statistical significance between PrP-siRNA and scrambled-siRNA groups. This result is consistent with those observed by Mallucci et al. [10], [11] showing down regulation of PrP^C^ expression using lentiviral expression vectors carrying PrP-shRNA. In this study, the lentivector carrying PrPshRNA was able to reduce PrP expression in mouse brains in the vicinity of the injection site, to rescue neuronal damages mainly in the area treated and to reverse the pathological phenotype in prion infected mice. Finally, we also observed an impact of PrP-siRNA on the glial reaction induced by prion infection. This effect was shown to be relevant since it was significant when compared to the scrambled-siRNA and the vehicle control groups.

The absence of statistical difference between PrP- and scrambled-siRNA treated groups for PrP^Sc^, vacuolization or the number of neurons, may be explained by an unspecific siRNA effect. Whether these observations result from a non-related response to siRNA molecules, whatever their sequence, or from a non-specific response due to the elevated proinflammatory state is unknown. Indeed, some animals of the scrambled-siRNA treated group exhibited high levels of inflammatory cytokines, notably IFN.

Improvement of the delivery strategy used here might be envisaged, notably on the time window of the treatment. Indeed, Mallucci et al. [10] and current research in progress in our group and collaborators [19],[21] have shown there is a “reversibility” window in which it is possible to rescue neuronal damages and clinical phenotypes. We have probably treated our mice too late during the course of the disease. Although the current results regarding the non-invasive treatment of mice with Aonys/siRNA delivery system are preliminary, they are encouraging since they show not only a decrease of PrP^C^ expression in the brain following a permucosal rectal route of administration but also an improvement of some neuropathological phenotype in our preclinical prion model.

## Materials and Methods

### Animal Model

Our study was approved by the “Comité régional Languedoc-Roussillon d’éthique en matière d’expérimentation animale” (UFR de Pharmacie, Avenue Charles Flahault 34060 Montpellier, France) under number CEEA-LR-1019. Mice were housed in an A3 facility according to the European Community Directive 86/609/EEC. C57Bl/6J female mice (from Charles Rivers laboratory) were anesthetized via intraperitoneal route with 100 µg/g of ketamine (Imalgène, Merial) and 5 µg/g of xylazine (Rompun, Baier). They were then intracranially inoculated with the ME7 prion strain (1%, 20 µl). Infected mice were observed every day for the appearance of prion related clinical signs. At this point, clinical signs were checked every day, and mice were finally sacrificed for ethical reasons when the progression of the disease was life threatening.

### Preparation of Aonys/siRNA Lipid Vector

PrP-siRNA (IBA GmbH, 5′ GUACCGCUACCCUAACCAATT 3′/3′ TTCAUGGCGAUGGGAUUGGUU5′) and scrambled-siRNA (5′ GACCCCAACUCCGAUAUCATT 3′/3′ TTCUGGGGUUGAGGCUAUAGU5′) were dissolved in nuclease-free water (Qiagen) and stored at −20°C until use. The lipid mixture Aonys was prepared extemporaneously by weighing the lipid constituents. At a well-defined ratio, the Aonys/siRNA lipid monophasic microemulsion forms rapidly by short vortex mixing of the lipid mixture with the siRNA solution.

### Treatment Delivery

The dosage forms were administered by rectal route using a micropipette and adapted conical tips. The possible rejection was documented and the quantity administered to each animal was adjusted according to body weight. A constant dosage-volume of 1 ml/kg was used. Animals were allocated in different groups (n = 12 per group) on day 90 and received 5 administrations/week, no administration during the weekend) of Aonys/siRNA by rectal mucosal deposit.

### Blood and Tissue Sampling

On month 5, blood of some animals (10 animals of each group) was collected by retro-orbital bleed using capillaries. Tubes were immediately centrifuged and the supernatant (plasma) was collected and kept on ice. Samples were stored at −80°C until analyses. Brains were removed and cut into 2 parts: half of tissue was fixed in PFA4% for histological and immuno-histochemical analyses and the other half was frozen at −80°C for biochemical analysis.

### Histological and Immunohistological Analysis

Brains were manually embedded in paraffin. Brain sections of 5 µm were then produced using a Microm microtome and collected on Strafrost glasses (Microm, France). Tissues were dewaxed using Clearify solution (American Master Tech Scientific, Inc) and then rehydrated with decreasing degrees of ethanol washes. For histological studies, samples were stained with Hematoxylin (Gill’s formula H-3401, Vector laboratories) for 3 minutes at room temperature, washed with water, and treated with acid ethanol. Samples were then washed with water and incubated with Eosin 2% during 3 minutes at room temperature and finally washed again. Slides were scanned using the (Nanozoomer Slide Scanner, Hamamatsu, platform MRI, INM Montpellier). Vacuole numbers and surface areas were determined (nb/mm^2^ and surface µm^2^/mm^2^) using ImagJ software.

#### GFAP and NeuN immunohistochemistry

Sections were pretreated with proteinase K (Roche, PK 20 µg/ml) for 10 minutes at 37°C for GFAP immunostaining or with EDTA buffer (1 mM) during 20 minutes in micro-wave for NeuN immunostaining. They were then washed with water and immersed in hydrogen peroxide 0.5% for 20 minutes at room temperature. After a saturation step (PBS-0.1%BSA-0.1%Triton X-100) for 1 hour, sections were incubated overnight at 4°C with the anti-GFAP (Dako) or NeuN primary antibody (1∶100 in PBS-0.1%BSA-0.1%Triton X-100). The secondary antibodies used were a biotinylated goat anti-rabbit antibody (Amersham) or a biotinylated goat anti-mouse antibody (vectors) (1∶1000 in PBS-0.1%triton X-100). The avidin-peroxidase complex (Vectastain Elite kit, Vector laboratories) was added and then revealed with 3,3′-diaminobenzidine (DAB) (Vector laboratories), according to the manufacturers’ instructions.

#### PrP^Sc^ immunohistochemistry

PrP^Sc^ was analysed by immunohistochemistry using the Saf84 (0.5 µg/ml) anti-PrP antibody [21]. SAF84 monoclonal antibody recognising the human 161–170 PrP sequence was kindly provided by Dr J. Grassi (CEA/SPI, Saclay, France). For PrPSc immunostaining, epitope retrieval consists in a treatment with formic acid (10 minutes) followed by an autoclaving treatments (121°C, 10 minutes). The secondary antibody used was a biotinylated goat anti-mouse antibody (Amersham, France) (1∶1000 in PBS-0.1%triton X-100). The avidin-peroxidase complex (Vectastain Elite kit, Vector laboratories, Clinisciences) was then added and then revealed with 3,3′-diaminobenzidine (DAB).

### Western Blot Analysis

After tissues homogenization and extraction, western blot were performed as previously reported [21], using SAF32 anti-PrP antibody for PrP^C^ and a mixture of SAF60, SAF69 and SAF70 after proteinase K digestion (200 µgPk/100 mg of tissue) for PrP^Sc^.

### ELISA

The enzyme immunometric assays (EIA) were performed according to the manufacturer recommendations (SPI-Bio). Tissue homogenates (10% in PBS) were diluted in the extraction buffer of the kit and analyzed. The SPI-Bio kit capture antibody was specific to prion protein residues 144–153 and the detecting antibody recognized the octapeptide-repeat region located in the N-terminal part of the PrP. Readout analysis was performed using TECAN, Xreadplus system (Tecan group).

### Pro-inflammatory Cytokine Quantification

RayBiotech kit (G series) glass chip format to simultaneously detect multiple cytokine expression levels was used. The signals from G series arrays were detected using a laser scanner. The following analytes were quantified in different serum samples from the mice of different groups: Eotaxin, Fas Ligand, bFGF, G-CSF, GM-CSF, IFN-gamma, IGF-II, IL-1 alpha, IL-1beta, IL-12 p40/p70, IL-12 p70, IL-13, IL-6, IL-9, Leptin, MCP-1, M-CSF, MIG, PF-4, TIMP-1, TIMP-2, TNF-alpha, Thrombopoietin, and VEGF.

### Statistical Analysis

Raw data were entered into calculation sheets (Microsoft Excel®). The mean and standard deviation were calculated for each group. Results in grouped data tables or figures are presented as means ± standard error of the mean (s.e.m.) or as medians and interquartile ranges. Medcalc (13.0) and PASW software (SPSS) were used for statistical analysis. The Wilcoxon test was also used to test the significance of the difference between two sample groups (equivalent to the Mann-Whitney test on unpaired observations). Significance was set at the p = 0.05 level and statistical power P>80%.
